# Comparative Genomic Analysis of *Holospora* spp., Intranuclear Symbionts of Paramecia

**DOI:** 10.3389/fmicb.2018.00738

**Published:** 2018-04-16

**Authors:** Sofya K. Garushyants, Alexandra Y. Beliavskaia, Dmitry B. Malko, Maria D. Logacheva, Maria S. Rautian, Mikhail S. Gelfand

**Affiliations:** ^1^Skolkovo Institute of Science and Technology, Moscow, Russia; ^2^Kharkevitch Institute for Information Transmission Problems, Moscow, Russia; ^3^Institute of Integrative Biology, University of Liverpool, Liverpool, United Kingdom; ^4^Department of Invertebrate Zoology, Faculty of Biology, Saint Petersburg State University, Saint Petersburg, Russia; ^5^National Research Center for Hematology, Moscow, Russia; ^6^Faculty of Bioengineering and Bioinformatics, Lomonosov Moscow State University, Moscow, Russia; ^7^National Research University Higher School of Economics, Moscow, Russia

**Keywords:** *Holospora*, *Rickettsiales*, endosymbionts, paramecia, ciliates, nuclear symbionts, genome

## Abstract

While most endosymbiotic bacteria are transmitted only vertically, *Holospora* spp., an alphaproteobacterium from the *Rickettsiales* order, can desert its host and invade a new one. All bacteria from the genus *Holospora* are intranuclear symbionts of ciliates *Paramecium* spp. with strict species and nuclear specificity. Comparative metabolic reconstruction based on the newly sequenced genome of *Holospora curviuscula*, a macronuclear symbiont of *Paramecium bursaria*, and known genomes of other *Holospora* species shows that even though all *Holospora* spp. can persist outside the host, they cannot synthesize most of the essential small molecules, such as amino acids, and lack some central energy metabolic pathways, including glycolysis and the citric acid cycle. As the main energy source, *Holospora* spp. likely rely on nucleotides pirated from the host. *Holospora*-specific genes absent from other *Rickettsiales* are possibly involved in the lifestyle switch from the infectious to the reproductive form and in cell invasion.

## Introduction

Symbiotic associations with bacteria are common to many, if not all, eukaryotes. Endosymbiotic bacteria are well studied in plants, insects, and some other organisms (Moran et al., 2005; Kawaguchi and Minamisawa, 2010; McCutcheon and Moran, 2010; Bennett and Moran, 2013; Becker et al., 2015; Wu et al., 2015; Wexler et al., 2016; Zipfel and Oldroyd, 2017). By the original definition (Oulhen et al., 2016), a symbiotic relationship does not imply benefits for the host. Nevertheless, in symbiotic relationships, symbiont explores its host as a habitat. In turn, host can acquire some necessary products and/or properties which it unable to synthesize, e.g., ammonium produced for plants by nitrogen fixing bacteria, essential amino acids produced by endosymbionts of sap-sucking insects (Moran et al., 2005; Archibald, 2015; Sabater-Muñoz et al., 2017), or vitamins for blood-feeding tsetse fly (Dale and Welburn, 2001). Some symbionts, like some strains of *Wolbachia* spp., protect the host from pathogenic viruses (Hedges et al., 2008); or are necessary for the maturation of the immune system, as e.g., *Wigglesworthia* for tsetse flies (Weiss et al., 2011). *Caedibacter* creates growth advantages for infected paramecia and helps them to out-compete uninfected ones (Kusch et al., 2000). An endosymbiont of ciliates from the genus *Euplotes* is essential for the host cell proliferation (Vannini et al., 2012, 2017). However, most relations are not well understood, and an advantage for the host is not always demonstrated, especially for facultative endosymbionts (Lu et al., 2016; Yañez et al., 2016).

Endosymbiotic bacteria and endonuclear parasites typically have several characteristic features, such as small genome size, low GC content, and short intergenic spacers (reviewed in McCutcheon and Moran, 2012; Batut et al., 2014; Martínez-Cano et al., 2015). Another important trait of these bacteria is an accelerated rate of genome evolution, caused by the minimal gene flow and substantial genetic drift due to the small effective population size of endosymbionts (Marais et al., 2008; Boscaro et al., 2017; Sabater-Muñoz et al., 2017). This leads to formation of pseudogenes and gene loss, and an overall decrease of the metabolic capacity (McCutcheon and Moran, 2012; Boscaro et al., 2017). The loss of genes involved in the reparation drives an even faster evolution of symbiotic species (McCutcheon and Moran, 2012). It also can lead to the decrease in the GC content (Horst et al., 1999; Moran et al., 2005; Long et al., 2018). A stable environment in the host cell does not require complicated regulatory systems, yielding reduction of intergenic regions (McCutcheon and Moran, 2012).

*Holospora* spp. are endonuclear symbiotic *Alphaproteobacteria* from the order *Rickettsiales* that inhabit either macro- or micronucleus of *Paramecium* spp. It has been suggested that *Holospora* spp. are parasites, as other *Rickettsiales*. A recent analysis of 16S rRNA lead some researchers to separate the basal rickettsial lineage of *Holospora*-like bacteria in a separate order *Holosporales* (Ferla et al., 2013; Szokoli et al., 2016), but for convenience we discuss both orders here together as *Rickettsiales*, because they have similar lifestyle and metabolic capacities. The genus *Holospora* currently is comprised of nine species, each showing clear nuclear and host specificity (Görtz and Schmidt, 2015). *Holospora* are not able to reproduce outside the host cell but can leave the host in order to invade a new one. Unlike most of the studied symbiotic species, *Holospora* have a complex life cycle involving two morphologically different forms. The short reproductive form exists only in the host nucleus and multiplies by binary fission, while the long infectious form does not multiply and is capable of infecting new host cells (Gromov and Ossipov, 1981; Fokin and Görtz, 2009). This form shows a unique cytological organization with a pronounced polarity (a large perinuclear space and a special tip), which seems to be of functional significance for the infection process. The infectious form enters new host via *Paramecium* food vacuole together with food bacteria, but escapes digestion, exits the vacuole and enters the cytoplasm followed by nuclear invasion. Two main mechanisms of this process have been proposed: *Holospora* either disrupt the digestive vacuole or enter the transport vacuoles (Schweikert et al., 2013). Then *Holospora* reach the nucleus by utilizing the host’s actin cytoskeleton (Fokin and Görtz, 2009; Sabaneyeva et al., 2009). In *Holospora obtusa*, the 89 kDa periplasmic protein was shown to be associated with cell invasion (Iwatani et al., 2005). The invasion of macronucleus by *H. obtusa* is associated with release of the 63 kDa periplasmic protein into the macronucleus of the host (Abamo et al., 2008). Additionally, 15 and 39 kDa periplasmic proteins are released from the cell tip during the macronucleus infection (Fujishima et al., 1997). Mechanisms underlying the transition from the reproductive to the infectious form and back are not well understood, but the 5.4 kDa protein with a signal peptide is only detected in the intermediate and infectious forms (Dohra et al., 1997).

In the presence of *Holospora*, *Paramecium* is able to grow, divide and mate (Schweikert et al., 2013). *Holospora* contribute to the heat-shock resistance in *Paramecium caudatum*, as cells infected with *H. obtusa* express high levels of hsp70 mRNA (Fujishima et al., 2005). They may also assist in acquiring high-salt and osmotic-shock resistance to the host (Fujishima, 2009). The presence of *H. caryophila* in the *Paramecium biaurelia* nucleus is advantageous in several cell lines during the exponential growth (Bella et al., 2016). Despite these observations of benefits for the host, *Holospora* have been shown to negatively affect host cells. For example, the presence of *Holospora elegans* in *Paramecium* leads to the formation of dysfunctional macronucleus during conjugation (Görtz and Fujishima, 1983). *Holospora undulata* increases mortality of the host, especially at low-food treatment (Restif and Kaltz, 2006), and high concentrations of the infectious form in the macronucleus inhibit the host cell growth (Fujishima, 2009). The relationship between *Holospora* spp. and their paramecia hosts seems to be a complex system in which benefits or damages for the host can be highly context-dependent.

The metabolism of *Rickettsiales*, and in particular of *Rickettsia* spp., has been studied in detail (Andersson et al., 1998; Hotopp et al., 2006; Fuxelius et al., 2007; Georgiades et al., 2011). *Rickettsia* export at least 51 metabolites from the host (Driscoll et al., 2017). They are not able to synthesize amino acids, nucleotides, lack glycolysis, and have to import such compounds as coenzyme A, pyruvate, FAD, biotin, etc.

So far, three *Holospora* genomes have been sequenced, all of which are endosymbionts of *Paramecium caudatum:* macronucleus-specific *H. obtusa* and micronucleus-specific *H. elegans* and *H. undulata* (Dohra et al., 2013, 2014). Analysis of common orthologous genes has yielded 572 single-copy core genes shared by the three genomes, and *Holospora* have been shown to rely on the host for energy production (Dohra et al., 2014). However, no detailed metabolic pathway reconstruction has been performed.

Here, we report a comparative analysis of four *Holospora* species, including *Holospora curviuscula*, a newly sequenced macronuclear endosymbiont of *P. bursaria.* We propose that *Holospora* use host-produced nucleotides as its energy source. We also identify the essential compounds that *Holospora* are likely able to synthesize, and compare their metabolism to that of other *Rickettsiales.*

## Materials and Methods

### Growth Conditions and Genomic DNA Preparation

*Holospora curviuscula* has an obligate association with its host, *Paramecium bursaria*, and is therefore uncultivable, so the bacteria have been grown inside host cells. *Holospora curviuscula* strain NRB217 from *Paramecium bursaria* isolated in the Novosibirsk Akademgorodok were obtained from the infected clones maintained in the CCCS (Culture Collection of Ciliates and their Symbionts), Research Park, Saint-Petersburg State University. The host cells were cultivated at the room temperature on the lettuce medium inoculated with *Enterobacter aerogenes* as a food resource for paramecia. The culture of *P. bursaria* bearing *H. curviuscula* was concentrated by centrifugation (10 min at 4500 *g*) and then homogenized using 10% solution of detergent Nonidet P-40 (Sigma-Aldrich Cat No. 21-3277 SAJ). The infectious forms of *H. curviuscula* were isolated from the homogenate by centrifugation in Percoll density gradient (Sigma-Aldrich Cat No. P1644) as described previously (Rautian and Wackerow-Kouzova, 2013). Genomic DNA was isolated with the DNeasy Blood and Tissue kit (QIAGEN Cat No. 69504) using a modified protocol — the time of incubation of cell homogenate with ATL buffer and proteinase K was increased to 16 h. All the subsequent steps were performed according to the standard Quick-Start Protocol.

### Genome Assembly and Annotation

Two libraries were generated, paired-end MiSeq Illumina library (2 × 250) (PE), and mate-pair library with insertion size 4 kbp (MP). The initial MP library size was 2.5 million reads, and the PE library size, 4.5 million. MP reads were filtered with NextClip v1.3 (Leggett et al., 2014) and only category A pairs of reads (both reads in a pair contain adapters) were selected for further analysis. Both MP and PE were filtered by quality with trimmomatic version 0.33 (Leading 15, sliding -15, slidingwindow -4:25), after filtering 1.3 million MP reads and 1.7 million PE reads were retained for assembly. Processed reads were assembled with platanus version 1.2.1. The Whole Genome Shotgun project has been deposited at DDBJ/ENA/GenBank under the accession PHHC00000000. The version described in this paper is version PHHC01000000.

To estimate the completeness of the *Holospora* assemblies, we used HMMer to search for essential PFAM domains (Rinke et al., 2013). A domain was considered to be present, if it was found by hmmsearch (with default parameters) (Mistry et al., 2013) and the bias was lower than the hit score. For all missing genes we checked whether they were present in 60 genomes of *Rickettsiales* and 23 other endosymbiotic *Proteobacteria* with tiny genomes less than 0.8 Mb (the complete list of genomes is given in Supplementary Table 1).

The resulting contigs were annotated with the PROKKA pipeline (Seemann, 2014). Metabolic pathways were reconstructed with SEED (Overbeek et al., 2005) and BioCyc (Caspi et al., 2016). To search for known bacteriocins we used APD3 (Wang et al., 2016), BAGEL3 (van Heel et al., 2013), and searched for known bacteriocin-associated PFAM domains. AntiSMASH (Weber et al., 2015) was used to predict biosynthesis of secondary metabolites. Transmembrane helices in proteins were predicted with TMHMM^1^. Transporter protein specificity was predicted by TCDB (Saier et al., 2016). SignalP and SecretomeP were used to predict signal peptides and secreted proteins, respectively (Bendtsen et al., 2005a; Nielsen, 2017). To predict the twin-arginine motif we utilized TatP server (Bendtsen et al., 2005b). Autotransporter proteins were predicted by search for PFAM domains PF03797, PF12951, PF05662, PF05658, PF11924, PF11557, PF16168, PF15403, PF03895 with HMMer package. To find phage-like regions in the genome assemblies, we used PHAST (Zhou et al., 2011). Additionally, we searched for all PFAM-domains with the keywords “transposase” and “phage” (328 PFAM domains).

### 16S rRNA-Based Phylogeny

To reconstruct the phylogenetic tree of *Holospora* spp., the PhyML software (Guindon et al., 2010) was used. 16S rRNAs from *H. curviuscula* NRB217, *H. elegans* E1, *H. obtusa*, and *H. undulata* HU1 were extracted from the full genome sequences, while 16S rRNAs from *H. acuminata* and *Candidatus* Gortzia infectiva were obtained from the SILVA database (accession numbers: KC164379 and HE797907, respectively). To root the tree, we used 16S rRNA of *Rickettsiales* (SILVA accession number: CP009217). The phylogenetic tree was constructed with PhyML v. 3.1 with 100 bootstrap replicates, GTR substitution model, and -o tlr parameter.

### Analysis of Repeats

To estimate the fraction of repetitive DNA in the *Holospora* genome assemblies, reads were realigned with Bowtie 2 (–end-to-end mode) (Langmead and Salzberg, 2012) back to the respective assemblies, and the coverage for each nucleotide was calculated. In the case of *H. curviuscula*, we used PE reads generated in this study, reads for *H. obtusa* and *H. undulata* were downloaded from DDBJ (accession numbers DRP001203 and DRA001008, respectively). A fragment was presumed to be duplicated if it was longer than 20 bp, and its coverage was at least 1.6-fold higher than the median contig coverage.

### Orthologous Groups and Genome Comparisons

To investigate the phylogenetic relations between *H. undulata* and *H. elegans*, pairwise genome alignments of all *Holospora* genomes were constructed with MAUVE (snapshot 2015-02-13) (Darling et al., 2010). Alignments of orthologous genes from the essential list (see above) were extracted from the MAUVE output, and all gaps were removed. The number of substitutions was calculated for each gene for each pair of genomes.

To investigate the gene repertoire of all *Holospora* and other *Rickettsiales*, we constructed groups of orthologous genes using OrthoMCL v. 2.0.9 (Li et al., 2003) with the MCL software v. 14-137. The output gene clusters were grouped in five subgroups using an *ad hoc* perl script which considered orthologs, co-orthologs and inparalogs. All *Rickettsiales* and other *Holospora* genomes (for the complete list see Supplementary Table 2) were downloaded from NCBI Genbank (Benson et al., 2013).

## Results

### *Holospora curviuscula* Genome

**FIGURE 1:**
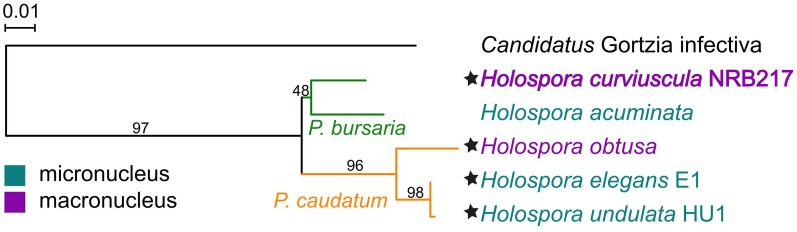
Maximum likelihood phylogenetic tree of *Holospora* spp. reconstructed by PhyML (see Materials and Methods). Violet, macronucleus endosymbiont; cyan, micronucleus endosymbiont. Genomes marked with stars have been sequenced.

The *H. curviuscula* draft genome sequenced here is comprised of 152 scaffolds (210 contigs) with N50 of ∼39 kbp, total length of 1.7 Mb, and GC content of 37.6% (**Table 1**). The longest scaffold is 153367 bp and contains 126 CDSs. We predicted 1594 genes, including 1555 protein-coding genes with the average encoded protein length of 218 aa (Supplementary Figure 1). We assigned protein function to 683 genes. The genome assembly contains all rRNA genes and a set of 36 tRNAs necessary to recognize all codons. The 16S rRNA analysis revealed that the sequenced genome indeed belongs to the genus *Holospora*, and specifically to *H. curviuscula*, and is closer to *Holospora acuminata*, another endosymbiont of *P. bursaria*, than to *Holospora* from *P. caudatum* (**Figure 1**). The *H. curviuscula* genome is the largest *Holospora* genome sequenced so far (**Table 1**). Its GC content is slightly higher than that of most other non-*Rickettsiales* obligate endosymbionts (McCutcheon and Moran, 2012), but is typical for *Rickettsiales* (Supplementary Figure 2).

**Table 1:** Results of genome assembly and annotation of *H. curviuscula*, and comparison with other *Holospora* species.

	*H. curviuscula*	*H. obtusa*	*H. undulata*	*H. elegans*
Genome (bp)	1715500	1334837	1402636	1268333
No. of contigs	210	91	203	152
GC content (%)	37.6%	35.2%	36.1%	36.0%
CDS	1594	1117	1224	1212

**FIGURE 2:**
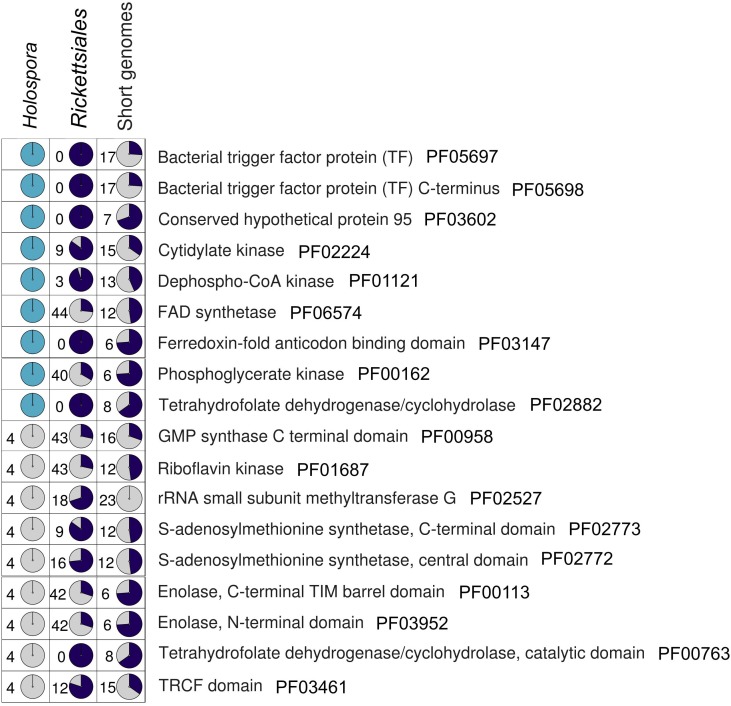
The presence/absence pattern of 18 essential PFAM domains in *Holospora*, endosymbiotic bacteria with tiny genomes, and *Rickettsiales* (Supplementary Table 1). Color code: dark blue, the domain is present; gray, the domain is absent; cyan represents domains found with decreased similarity threshold (see Materials and Methods). The number in each cell is the number of genomes where the domain has not been found.

We estimated the quality of the assembly by searching for nearly universal bacterial genes (see Materials and Methods). Among the 139 universal PFAM domains, 121 are present in the *H. curviuscula* genome. Nine of the 18 missing domains have been found with a lower similarity threshold, which makes their presence uncertain. The remaining nine of the 18 missing domains are absent in all other *Holospora* as well. Moreover, these domains are also absent either in some endosymbiotic bacteria with small genomes or in some *Rickettsiales* genomes (**Figure 2**), meaning that their absence can be tolerated. We further searched for these 18 missing domains in unassembled sequencing reads of *H. curviuscula* (14% of all reads), and found no indication of their presence.

We manually checked 615 ORFs for frameshifts, and found only nine ORFs with frameshifts. This number may be an underestimate, because *H. curviuscula* genome contains short ORFs with unknown functions that have no homologs outside *Holospora* and can be remnants of some ancestral genes. Still, it implies that the *H. curviuscula* genome contains few pseudogenes.

*Rickettsiales* have been reported to have an atypical rRNA operon (Andersson et al., 1998). However, all *Holospora* have a standard rRNA operon, with 23S and 16S genes located close to each other and separated by several tRNA genes.

### Reanalysis *H. undulata* and *H. elegans* Genomes

**FIGURE 3:**
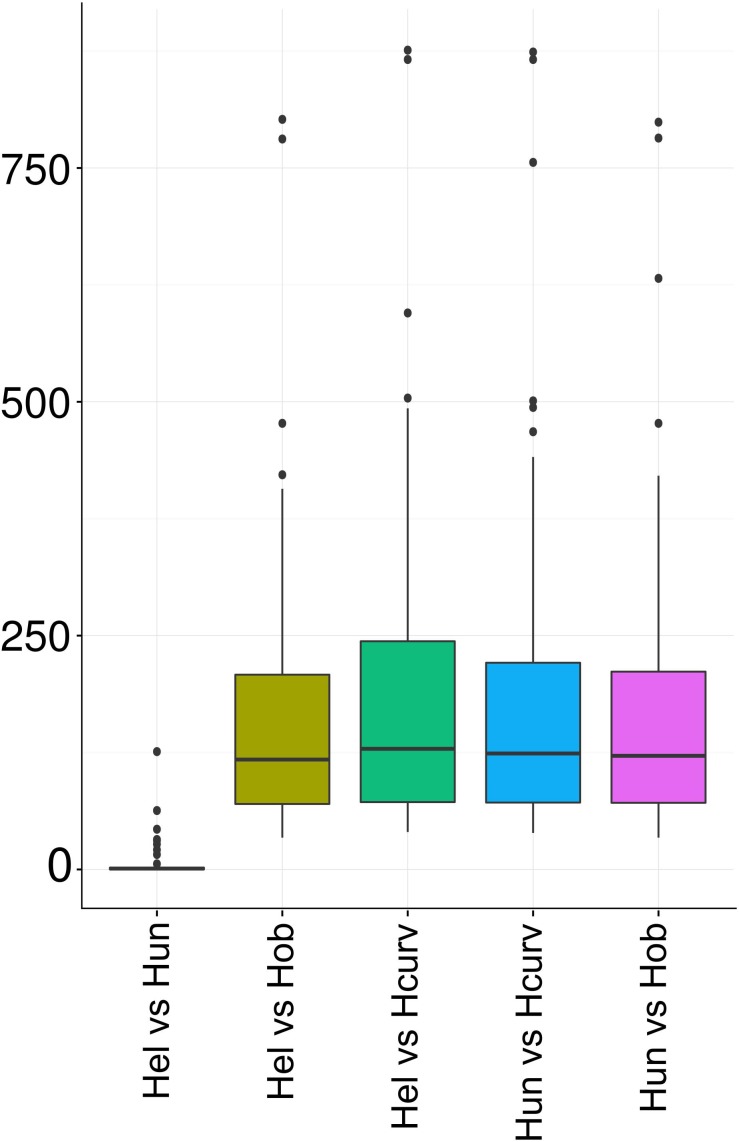
The number of mismatches in pairs of *Holospora* species. Hcurv, *H. curviuscula* NRB217; Hel, *H. elegans* E1; Hob, *H. obtusa* F1; Hun, *H. undulata* HU1.

*Holospora undulata* HU1 and *H. elegans* E1 extracted from the micronucleus of *P. caudatum* have been recognized as separate species based on differences in the cell morphology (Görtz and Schmidt, 2015). However, our comparison of 16S rRNA genes from the available genome assemblies (Genbank IDs NZ_ARPM00000000 and NZ_BAUP00000000, respectively) of these species revealed just a single-nucleotide difference (**Figure 1**), yielding > 99.9% sequence identity, which is above the common thresholds of 97 or 98.7% used to define species (Janda and Abbott, 2007). That led us to inquire whether the genomes of *H. undulata* and *H. elegans* indeed represented different species or just strains of the same species. We constructed whole-genome pairwise alignments of the *Holospora* genomes and counted mismatches in the essential genes (**Figure 3**). The *H. elegans*–*H. undulata* pair carried ∼100-fold fewer mismatches than all other genome pairs.

### *Holospora* spp. Have Multiple Repeat Sequences and Contain Prophages

**FIGURE 4:**
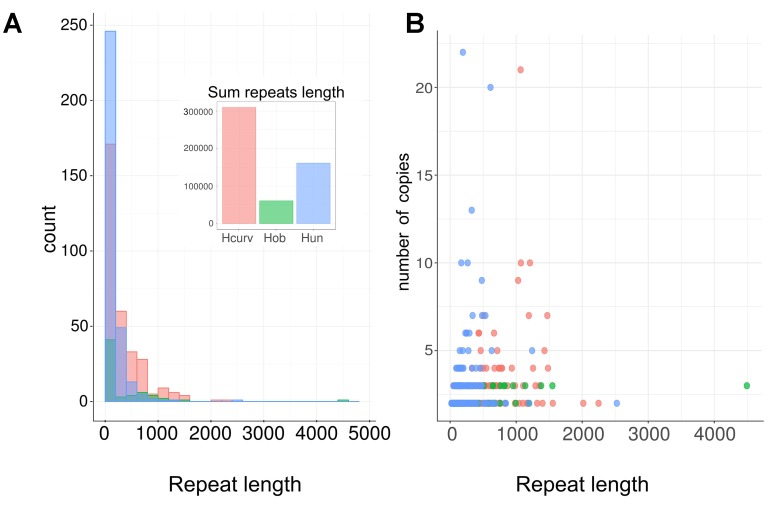
Lengths and the copy number distribution of repeats in *Holospora* spp. **(A)** Lengths of repeated sequences in *Holospora* spp. **(B)** Relations between repeat the repeat copy number and length. Hcurv, *H. curviuscula* NRB217 (pink); Hob, *H. obtusa* F1 (green); Hun, *H. undulata* HU1 (blue).

All available *Holospora* assemblies are comprised of multiple contigs, and reassembly has not led to better assemblies even with added genome coverage. Moreover, the genome length of *H. curviuscula* has been estimated at ca. 2 Mb (unpublished data), while the total assembly length is around 1.7 Mb. This suggests that *Holospora* genomes may contain a considerable fraction of repetitive DNA. To test this, we searched for fragments with high read coverage (see Materials and Methods). In *H. curviuscula*, we found ∼300 repeats with the total length of ∼300 kb (**Figure 4A**) and the estimated copy number varying from 2 to 21 (**Figure 4B**). In *H. obtusa* and *H. undulata*, the similarly estimated lengths of repetitive DNA were 60 and 161 kb, respectively. We were not able to estimate the fraction of repetitive DNA for *H. elegans*, as no raw sequencing data were available. The calculated repeat lengths could be underestimates, as we have applied strict coverage cutoffs yielding conservative repeat boundaries. For the same reason, we could overestimate the number of repeats by splitting long repeats into individual repeats in regions of low coverage. Still, the match between the difference in the assembly length and the genome length and the total coverage of repetitive DNA suggests that unassembled repeats comprise ∼15% of the genome in *H. curviuscula*. The high copy-number repetitive regions in *H. curviuscula* include short ORFs of possible transposases. In addition, the identified repeats include two copies of rRNA operons with the similar structure, and also genes encoding prophage-like proteins.

To characterize prophages further, we searched for complete prophages and, additionally, for phage-related PFAM domains in all *Holospora* genomes. We found two possible prophage regions in *H. elegans*, one in *H. obtusa*, and two in *H. undulata.* One of the *H. undulata* prophages was intact, contained all proteins necessary for the phage replication and was surrounded by integration sequences (Supplementary Figure 3); the remaining copies were severely disrupted and lacked insertion sequences. Although no intact prophages were found in *H. curviuscula*, scaffold565 had a locus with two DDE superfamily endonucleases and two putative transposases, and other scaffolds carried genes encoding phage capsid and phage portal proteins similar to the ones found in other *Holospora* as well as some full-length and fragmentary transposases (data not shown).

### *Holospora* spp. Cannot Synthesize Amino Acids and Some Other Essential Small Molecules

*Holospora* have a reduced metabolism even by the standards of the already gene-poor order *Rickettsiales* (Georgiades et al., 2011). In particular, *Holospora* lack genes encoding enzymes of the citric acid cycle (CAC) (Supplementary Figure 4). By contrast, *Rickettsia* and other *Rickettsiales* have at least some of these enzymes and use them to convert small molecules (Driscoll et al., 2017).

**FIGURE 5:**
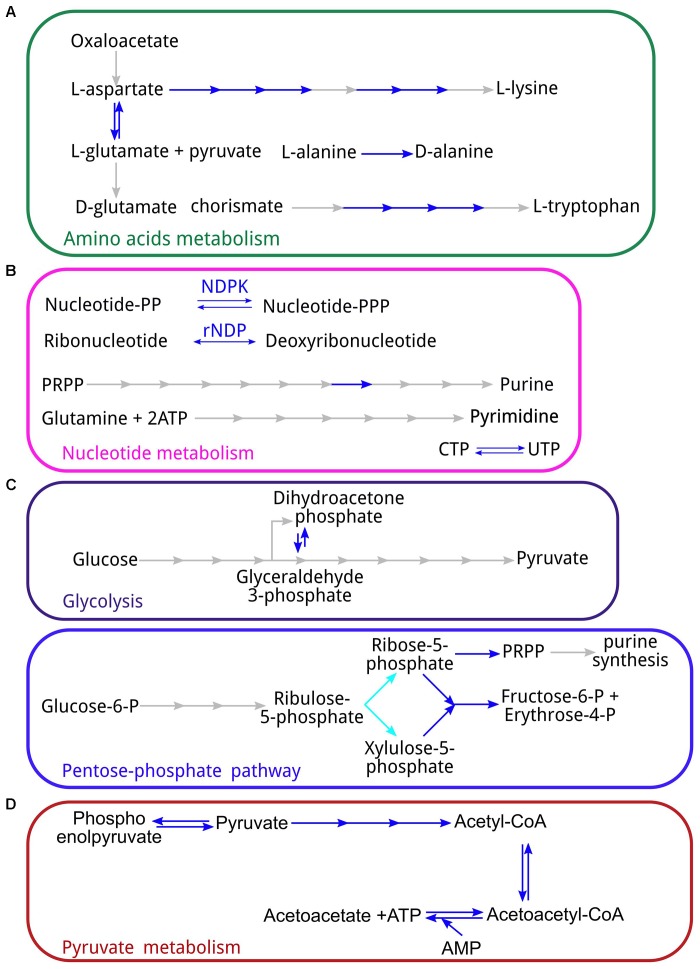
Some aspects of the *Holospora* metabolism: **(A)** amino acid metabolism; **(B)** metabolism of purines and pyrimedines; **(C)** energy metabolism (top – glycolysis, bottom – pentose phosphate pathway); **(D)** pyruvate metabolism. Blue arrows represent reaction for which the corresponding enzyme has been found in all *Holospora* spp.; cyan – enzymes absent in *H. curviuscula*, but present in *H. undulata* and *H. obtusa*. PRPP, phosphoribosyl pyrophosphate; NDPK, nucleoside-diphosphate kinase; rNDP, ribonucleotide reductase.

All sequenced *Holospora* lack amino acid synthesis pathways. For most amino acids, all enzymes of the pathway are missing. The exceptions are the partial pathways for the biosynthesis of L-tryptophane, L-lysine, and L-glutamate and for the conversion of L-alanine to D-alanine, an essential compound for formation of the cell wall (**Figure 5A**). This means that all amino acids have to be imported from the host. While *Rickettsia* also cannot produce amino acids and probably import them from the host (Driscoll et al., 2017), other *Rickettsiales* are capable of synthesizing some amino acids such as L-lysine and L-glutamine (Fuxelius et al., 2007). Similar to *Rickettsia, Holospora* cannot produce chorismate or use it as a substrate for downstream reactions, and need to import it or its derivates from the host.

Like *Rickettsia, Holospora* are not capable of purine and pyrimidine synthesis (**Figure 5B**), although, also like *Rickettsia*, they can convert UTP to ÑTP. Therefore, they need to obtain all nucleoside triphosphates from the host. On the other hand, *Holospora* carry genes for ribonucleotide reductases and therefore can convert ribonucleotides into deoxyribonucleotides.

The ubiquinone biosynthesis pathway is also partial, as it is comprised of only dimethylallyl diphosphate (DMAPP) and the enzymes downstream of it (Supplementary Figure 6), similarly to what has been reported for *Rickettsia* (Driscoll et al., 2017). Unlike *Rickettsia, Holospora* are unable to convert DMAPP to isopentenyl diphosphate, although they carry geranyl-diphosphate synthase which is missing in *Rickettsia*. Therefore, DMAPP seems to be obligatorily imported from the host.

The only major metabolic pathway that is almost intact is the fatty acids synthesis (Supplementary Figure 5).

In order to determine how the missing compounds are imported from the host, we analyzed predicted transporters. In *H. curviuscula*, thirty transport-associated genes have been identified. As expected, *Holospora* genomes encode oligopeptide and amino acid transporters, as well as proteases, some of which are periplasmic. In particular, we have found arginine/glutamine, proline, acetyl-serine/cysteine, choline/glycine/betaine, and putative branched amino acids candidate transporters. In addition, we found putative transport systems for magnesium, ferric ions, ribose, purines, sulfoacetate, L-galactonate or other sugars, and putrescine. However, these transport systems are insufficient to deliver all missing compounds to *Holospora*; in particular, it is not clear, how the remaining amino acids are delivered, unless as components of oligopeptides.

### *Holospora* spp. Use Nucleotides as the Main Energy Source

All *Holospora* lack most genes involved in energy production. In particular, all enzymes necessary for the glycolysis except phosphoglycerate mutase (**Figure 5C**) and all enzymes of the citric acid cycle except malate dehydrogenase (Supplementary Figure 4) are missing. The F_1_F_0_-ATPase is also missing. In addition, *Holospora* cannot produce coenzyme A from scratch, although as *Rickettsia*, they seem to have CoaE (PF01121, **Figure 2**) and thus are able to synthesize coenzyme A from dephospho-CoA (Driscoll et al., 2017). Of the pentose phosphate pathway, only the non-oxidative branch is present, as well as the downstream enzyme ribose-phosphate mutase that converts phosphorybosil pyrophosphate (PRPP) to sugars (**Figure 5C**); the energy-producing oxidative pathway leading to the ribulose-5-phosphate is missing. By contrast, all *Holospora* have the pyruvate dehydrogenase complex and are able to convert pyruvate to acetyl-CoA, and further to acetoacetyl-CoA and acetoacetate, with the production of ATP (**Figure 5D**). All *Holospora* have a set of ribonucleotide reductases, which would allow them to use either nucleotides or ribonucleotides as an energy source. No obvious source of energy other than nucleotides was found.

### Secretory Systems and Putative Invasins

Although most *Rickettsiales* are parasites and their genomes are highly reduced, they retain secretory systems such as the Tat and Sec pathways, a type IV system, and the TolC protein from a type I secretion system (Gillespie et al., 2015). Moreover, the VirB protein from a type IV secretory system is thought to be essential for the host invasion in most *Rickettsiales* (Rennoll-Bankert et al., 2015; Gillespie et al., 2016). By contrast, all studied *Holospora* demonstrate a significant decay of secretion pathways. They still possess the complete Sec system, additional systems helping to translocate proteins to the outer membrane (LolA, LolD, and possibly LolE) or transport them outside the cell (BamA, BamB, BamD, and chaperone DegP), and a TolC-like protein. However, we found no proteins similar to components of the Tat-system (TatA, TatB, or TatC). Since Tat-system proteins may be hard to identify (Gillespie et al., 2015), we performed a genome-wide search for proteins with the twin-arginine signal required for the recognition by the Tat-system and found no significant hits. Together, these observations show that the Tat transport system is indeed missing, which means that *Holospora* are unable to export folded substrates.

In *Rickettsia*, invasion is associated with proteins RalF, RickA, and Sca (Gillespie et al., 2015) which are missing in *Holospora*, implying that *Holospora* use other mechanisms to invade the host cell. There are also no proteins with ankyrin domains assumed to play a role in the pathogenesis in *Rickettsia* (Gillespie et al., 2015). Although Sec proteins are present in all *Holospora*, only one protein with a putative autotransporter domain (PF03797) was found in *H. curviuscula* (HCUR_00103). Even though this gene has homologs in other *Holospora*, this domain was not predicted in them, implying that it can be a false positive. However, we found multiple (3–9 copies per genome) genes similar to *ompA* in all *Holospora*. OmpA has been previously shown to be involved in the pathogenesis by *Rickettsiales* (reviewed in Palmer and Noh, 2012).

### *Holospora*-Specific Genes

**FIGURE 6:**
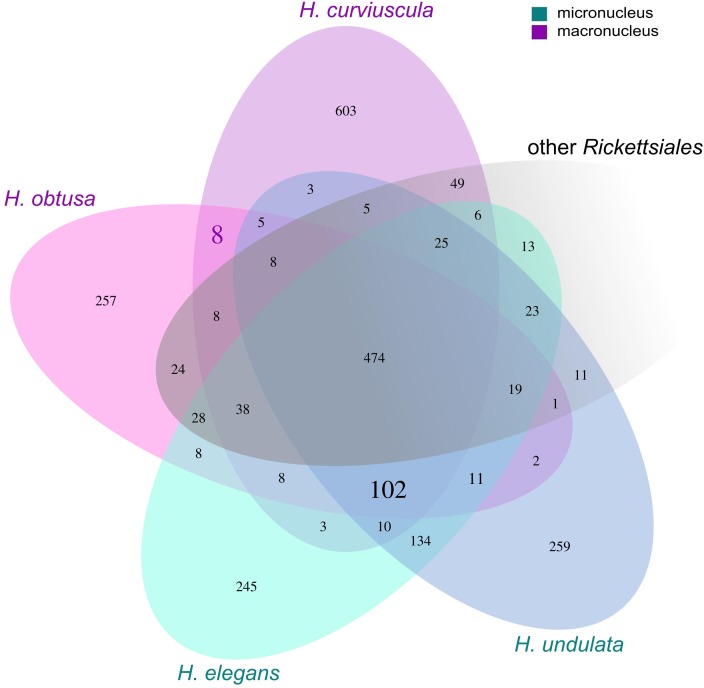
Groups of orthologous proteins in *Holospora* spp. and *Rickettsiales.* Numbers in cells indicate either the number of orthologous groups, or, in the case of unique genes, the number of singletons. Numbers in bold represent groups of orthologous groups common for either macronuclear (8) or all (102) *Holospora*. OGs common for the macronuclear endosymbionts are two OGs encoding alpha/beta hydrolases; one, a possible aspartate/glutamate racemase or a malate isomerase; one, nucleotide sugar epimerase; one OG had an unknown function; and three remaining OGs encode short proteins, of which one was similar to the DDE superendonuclease, and another one has a predicted signal peptide. Of 102 *Holospora*-specific OGs: 30 are secreted, 37 contain putative transmembrane helices; 17 HOGs contain short peptides, of which 5 are potentially secreted.

To search for genes that could be essential for the survival in the nucleus, we compared orthologous groups (OGs) for all *Holospora* with a set of all complete *Rickettsiales* genomes (**Figure 6**). We identified 102 OGs that occur in all *Holospora* and do not occur in any other *Rickettsiales* species (hereafter, *Holospora*-specific OGs, HOGs), 97 of HOGs contained no paralogs. HOGs encoded transporters, regulators, and hypothetical proteins. We expect proteins essential for survival and adaptation to the host either to be located on the surface of the bacterial cell or to be secreted. Therefore, we focused on those proteins that contained transmembrane helices or signal peptides. Thirty seven HOGs contained proteins with predicted transmembrane helices; 12 other OGs, proteins with predicted classic signal peptides; eighteen OGs were predicted to be secreted by the non-classical pathway (Supplementary Table 3). One of the latter HOGs contained the 89 kDa periplasmic protein which has been reported to be associated with cell invasion in *H. obtusa* (Iwatani et al., 2005). One HOG contained proteins similar to OmpA. These proteins are highly conserved among *Holospora*, and all *Holospora* except *H. curviuscula* carry two paralogs of this gene.

*Holospora* genomes encode numerous short (<100 aa) peptides with unknown functions. Short proteins have been shown to be involved in spore formation, regulation of transport, regulation of transcription, and signal transduction, or to possess antimicrobial or other toxic activities (Wang et al., 2008; Storz et al., 2014). Of the 102 HOGs, seventeen contained short proteins, including five HOGs that contained proteins with secretory signal peptide sequences. One of these HOGs has been described earlier as the 5.4 kDa protein involved in the switch between the reproductive and infectious forms (Dohra et al., 1997).

To determine whether there are proteins that could determine the nuclear specificity of Holospora, we searched for HOGs specific to the two macronuclear species, H. curviuscula and H. obtusa. We found eight HOGs present in both H. curviuscula and H. obtusa and absent in H. undulata, H. elegans and in other studied Rickettsiales. Of these, two HOGs encoded alpha/beta hydrolases; one, a possible aspartate/glutamate racemase or a maleate isomerase; one, nucleotide sugar epimerase; one HOG had an unknown function; and three remaining HOGs encoded short proteins, one of which was similar to DDE superendonuclease, and another one had a predicted signal peptide.

## Discussion

Here we report a comparative analysis of the newly sequenced genome of *H. curviuscula* NRB217 and other available *Holospora* genomes. The phylogenetic analysis of all available *Holospora* has confirmed (Rautian and Wackerow-Kouzova, 2013) that they are clustered by the host species rather than by micro- vs. macronuclear specificity, so that *H. curviuscula* is the outgroup to the previously analyzed genomes. Furthermore, the fact that we found only one substitution in the 16S rRNA gene and few substitutions genome-wide between the sequenced samples of *H. elegans* and *H. undulata* suggests that these are in fact strains of the same species.

The previous genomic analysis of *Holospora* spp. (Dohra et al., 2014) included only a general description of the available COG categories and some reconstruction of the metabolism. It has been observed that many pathways are missing in *Holospora*. The pyruvate dehydrogenase complex that is present in *Holospora* has been proposed to be a possible relic of an ancestral pathway. Additionally, it has been suggested that *Holospora* strongly relies on the host for energy production.

Addition of *H. curviuscula* allows for a detailed comparative genomic analysis of the metabolism of *Holospora* spp. We found that all *Holospora* have reduced metabolic capacities and have to import many metabolites from the host.

Even though the genomes of *Holospora* are relatively large (**Table 1**) in comparison with other symbiotic species such as *Buchnera*, *Candidatus* Baumannia, or *Candidatus* Carsonella, *Holospora* are unable to produce most of the essential compounds. Furthermore, all available *Holospora* genomes seem to contain a large fraction of repetitive sequences, which complicates the genome assembly. Arguably, as all available *Holospora* genomes consist of multiple contigs, some of the enzymes could have been missed. However, quantitative analysis suggests that all universal genes are indeed present and that nearly all non-repetitive regions are contained in contigs; any potential missing part of the genome would have to be small, and cannot account for a large number of missing genes. Furthermore, our analysis is based on independent assemblies of multiple moderately related species, and it is unlikely that the same gene would be missing in several assemblies.

While smaller endosymbionts can produce at least some amino acids or retain some parts of the central metabolism, *Holospora* are unable to synthesize any amino acid, and have to acquire them from the host, despite the fact that some relics of pathways are seen, such as a partial pathway for the tryptophan synthesis. *Holospora* lack glycolysis, the Entner-Doudoroff pathway, and the pentose phosphate pathway. Surprisingly, *Holospora* have no enzymes of the citric acid cycle, which is unusual for *Rickettsiales*, as even the most reduced *Rickettsia* retain it.

Although we have not performed a complete metabolic reconstruction, it is evident that a broad range of compounds, including amino acids, DMAPP, and chorismate derivates have to be imported from the host for *Holospora* to survive. It is unlikely that these compounds are accumulated by *Holospora* during its brief residence outside the nucleus. Indeed, the process of *Holospora* infection is a rapid one, whereas *Holospora* stay in the nucleus for long periods of time, which means that all these compounds have to be acquired from the host. Amino acids can be obtained by degrading host nuclear proteins, and indeed the *Holospora* infection is associated with increased proteolytic activity in the macronucleus (Freiburg, 1985). The situation with other compounds is more intriguing. All these small molecules can passively diffuse through the nuclear pore complex (Knockenhauer and Schwartz, 2016). Further, *Holospora* alter the host’s gene expression and increase RNA synthesis (Freiburg, 1985; Fujishima, 2009). These alterations may help bacteria to acquire the necessary nutrients. To understand how the missing nutrients are delivered to *Holospora*, we searched for transport proteins and attempted to predict their specificity. This has explained some, but not all of the missing nutrients, and this topic has to be investigated further.

We propose that *Holospora* not only relies on the host for the energy production, but specifically use nucleotides or ribonucleotides as the energy source. Indeed, they are able to interconvert them and to convert UTP to CTP, and putative ribose transport proteins, which can also transport nucleotides, are present. We suggest that ribonucleotides are the preferred energy source, as the intracellular abundance of ribonucleotides can be 10- to 100-fold higher than that of dNTPs (Traut, 1994). This seems natural, given the nuclear habitat of *Holospora*.

The interactions between *Holospora* and their hosts have been studied in some depth. *Holospora* infection can either decrease or increase the *Paramecium* viability under a variety of conditions (Fujishima et al., 2005; Hori et al., 2008; Fujishima, 2009; Bella et al., 2016). Addition of *Holospora curviuscula* to the analysis allowed us not only to investigate proteins specific to *Holospora* in a particular host, but also to find proteins that may play a role in the macronucleus infection. While the details of the mechanism remain unclear, it is likely that the interaction and stable infection rely on *Holospora*-specific proteins, in particular, secreted short conserved peptides. The 89 kDa periplasmic protein shown to be involved in the *H. obtusa* infection (Iwatani et al., 2005) is conserved in all studied *Holospora* spp., which indicates its importance. Finally, we propose that OmpA-like proteins may be involved in the *Holospora* invasion.

Overall, the analysis of the *Holospora* genomes demonstrates that their metabolic capabilities are unusually restricted, especially given the genome size, and provides a list of candidate genes essential for their unique lifestyle.

## Author Contributions

SG, MR, and MG designed the research. AB and MR isolated and cultivated bacteria. ML prepared the sequencing libraries and sequenced the genome. ML and SG assembled the genome. SG, AB, DM, and MG annotated the genome and performed the comparative analysis. SG, AB, MR, and MG wrote the paper.

## Conflict of Interest Statement

The authors declare that the research was conducted in the absence of any commercial or financial relationships that could be construed as a potential conflict of interest.

## Footnotes

1 http://www.cbs.dtu.dk/services/TMHMM/
