# Supplementary material for: Comparative Genomic Analysis of Holospora spp., Intranuclear Symbionts of Paramecia
Source: Front Microbiol. 2018 Apr 16;9:738. doi: 10.3389/fmicb.2018.00738 (PMC5911502; doi:10.3389/fmicb.2018.00738)
Supplement: Supplementary file 4 [file Data_Sheet_1.PDF]

Supplementary Figures

Comparative genomic analysis of *Holospora* spp., intranuclear symbionts of paramecia

Sofya K. Garushyants, Alexandra Ya. Beliavskaya, Dmitry B. Malko, Maria D. Logacheva, Maria S. Rautian, Mikhail S. Gelfand

Supplementary Figures 1-6

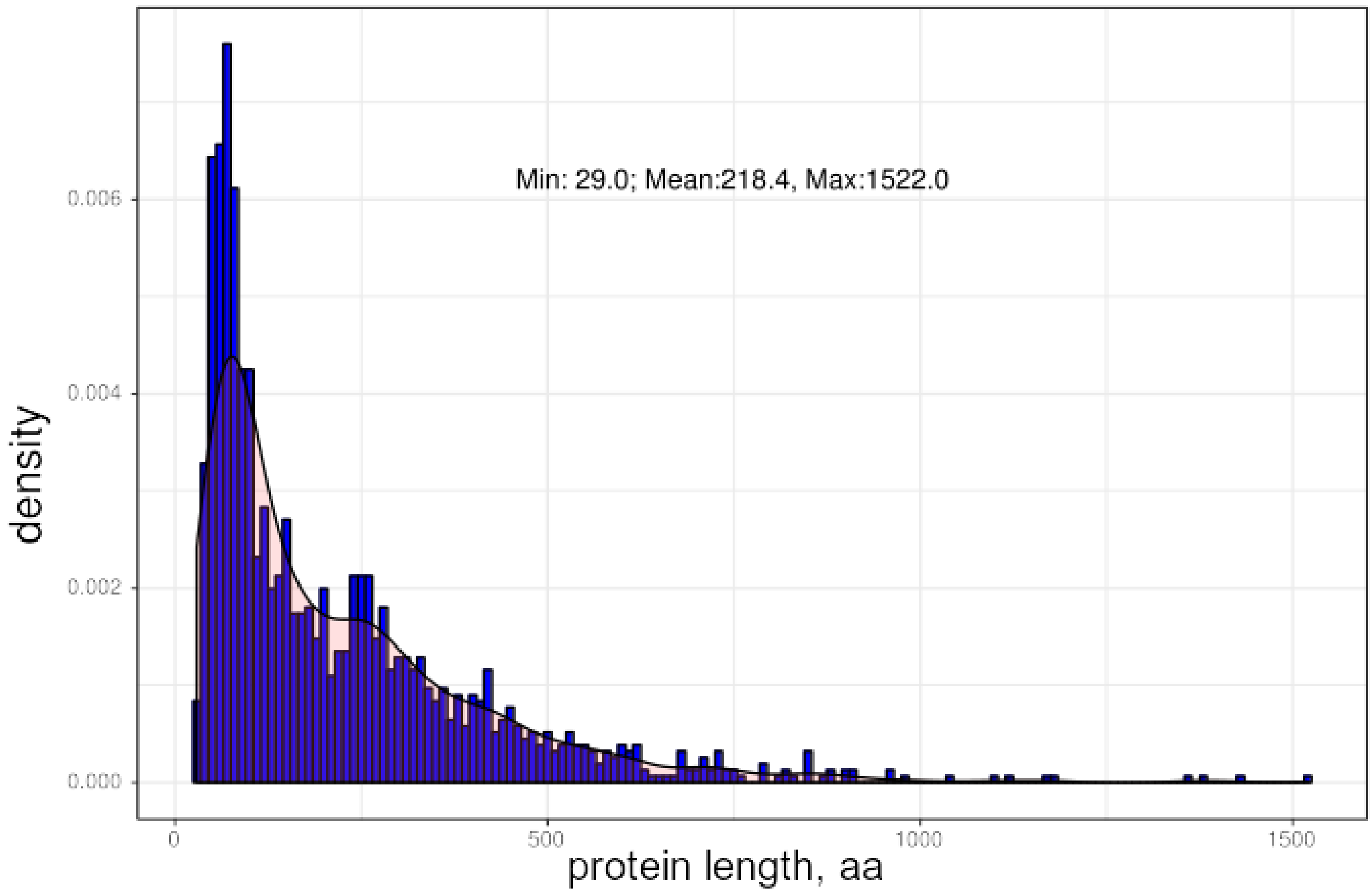

Supplementary Figure 1. Distribution of protein lengths in the *Holospora curviuscula* 02AZ16 genome.

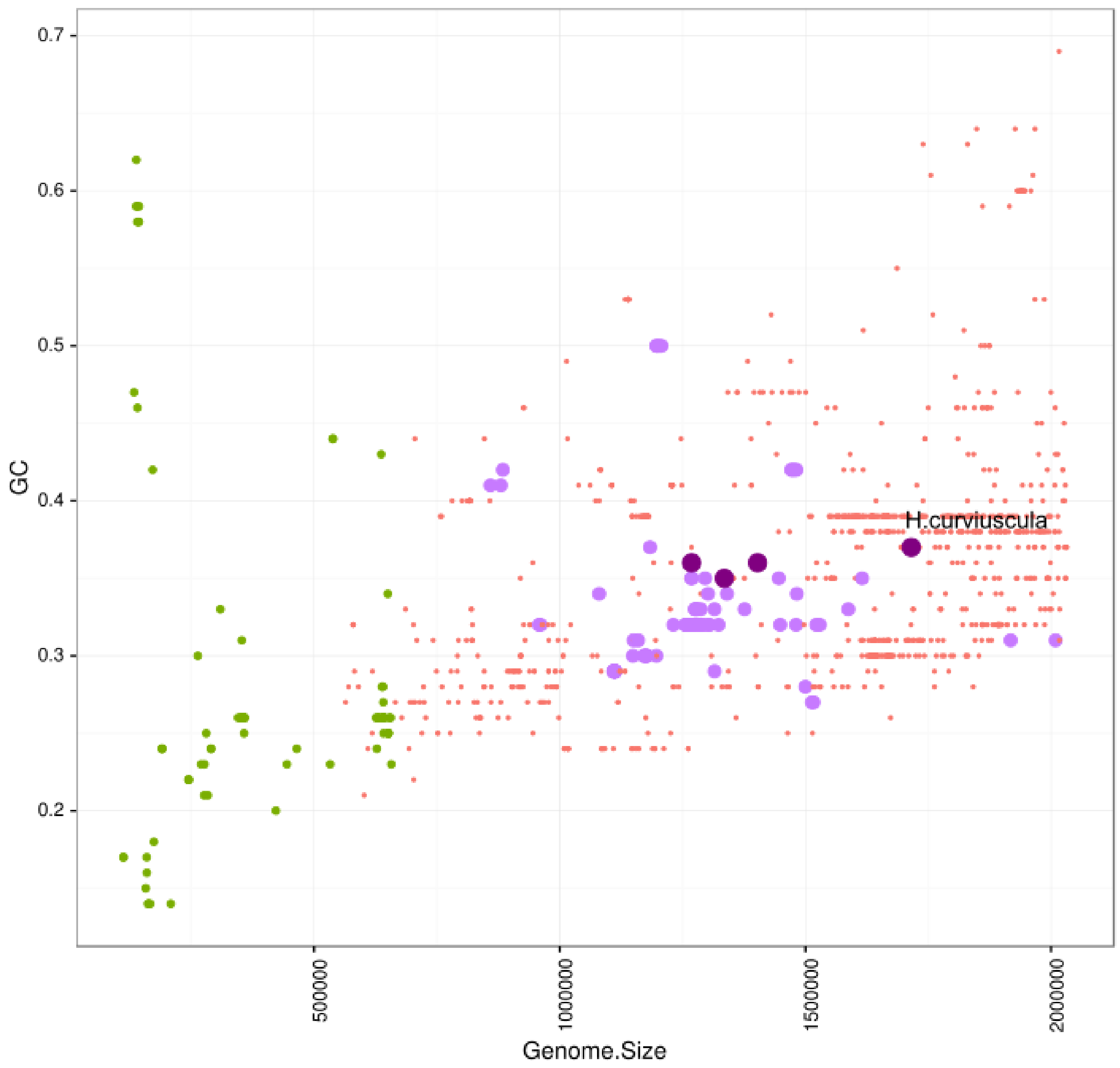

Supplementary Figure 2. Bacterial genome size versus genome GC-content. Each dot represents one genome. Colors: green – tiny insect endosymbionts, purple – Rickettsiales, dark purple – *Holospora* spp., pink – other bacteria.

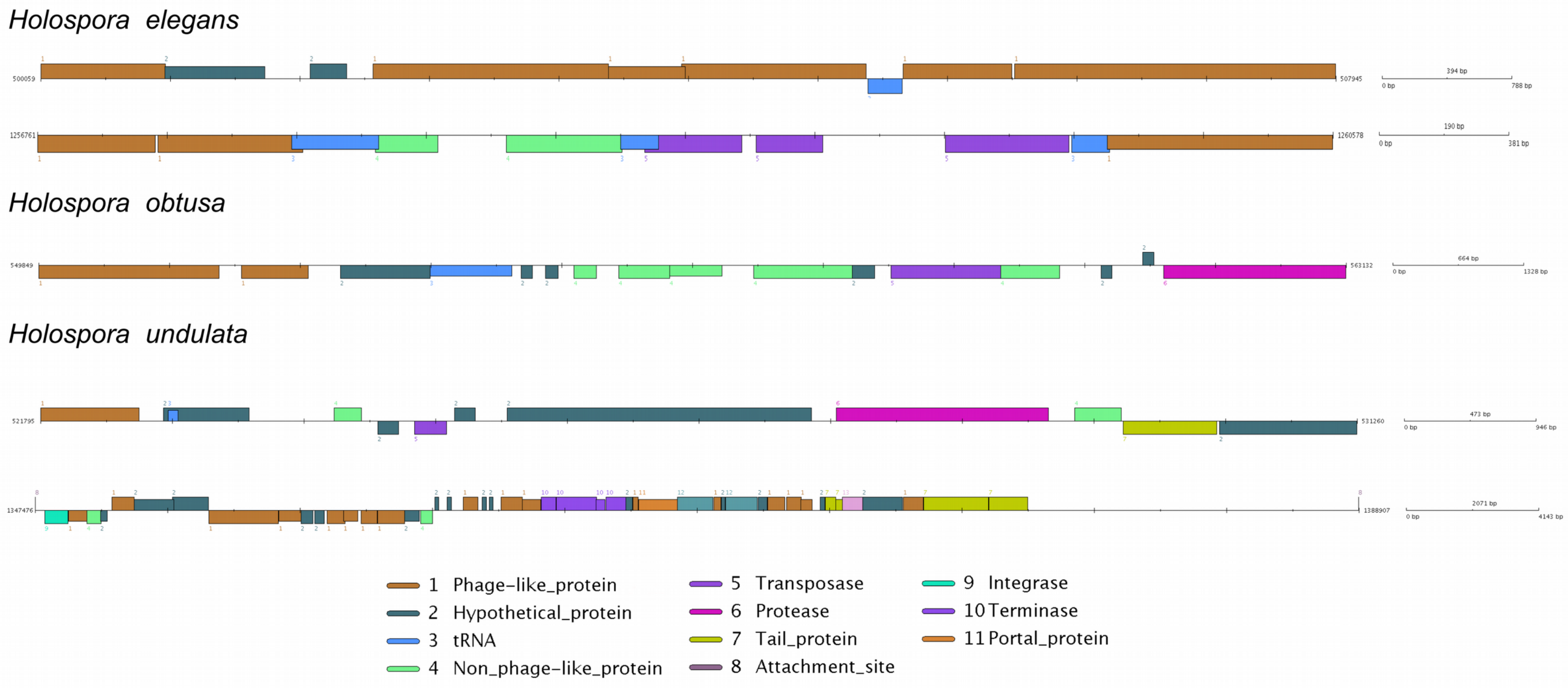

Supplementary Figure 3. Predicted by PHAST phage-like regions in *Holospora* spp.

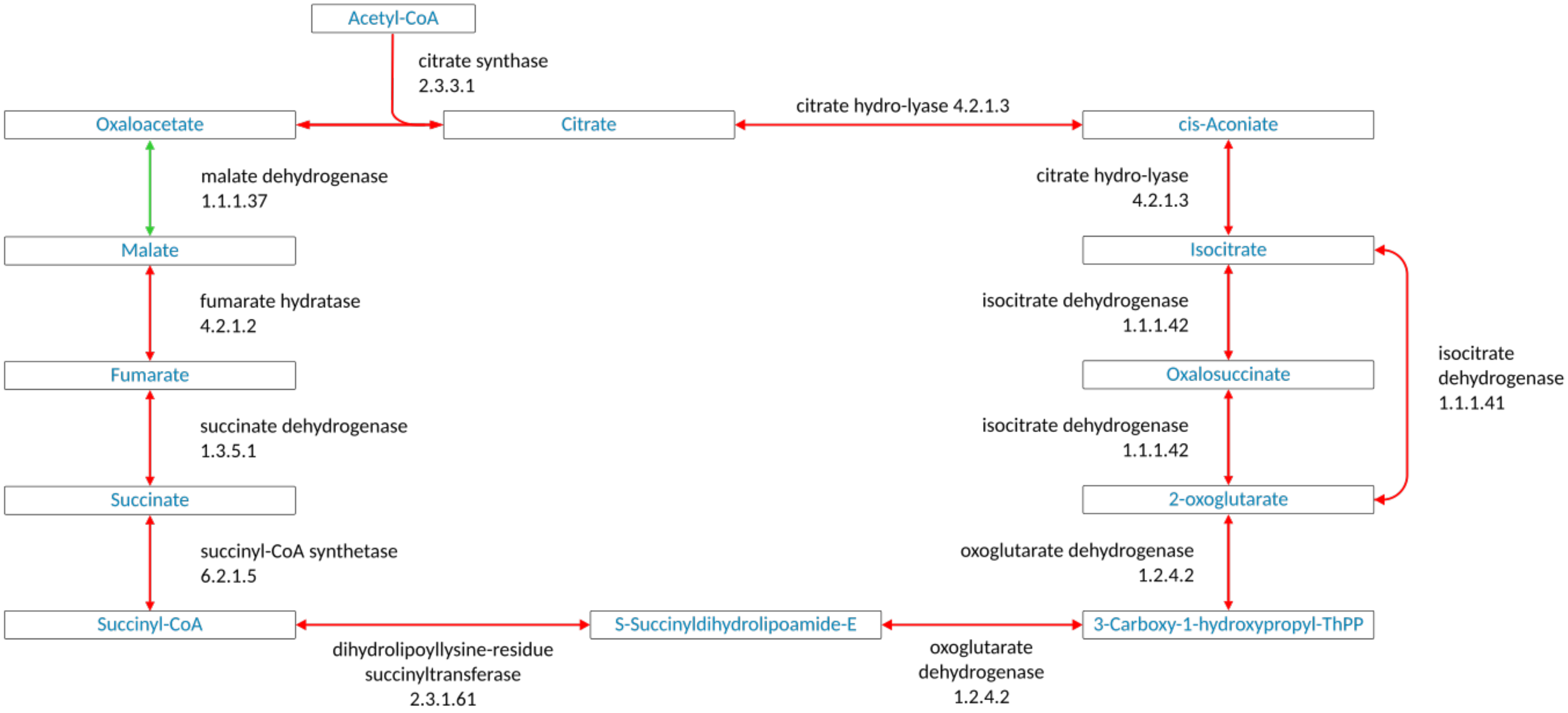

Supplementary Figure 4. The tricarboxylic acid (TCA) cycle in *Holospora* spp. Enzymes for all reactions are missing (red arrows).

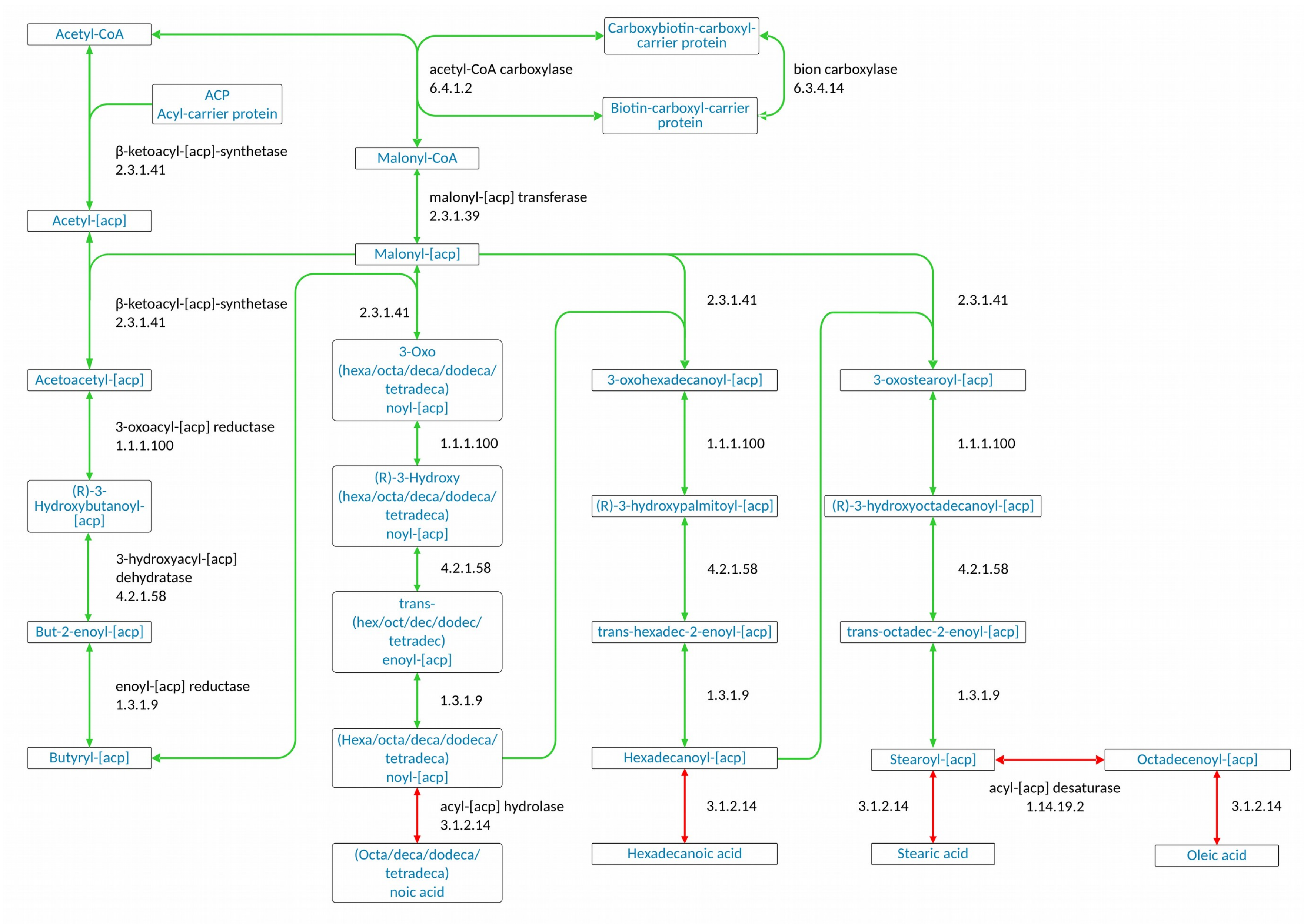

Supplementary Figure 5. Fatty acid metabolism in *Holospora* spp. Red arrows represents missing enzymes.

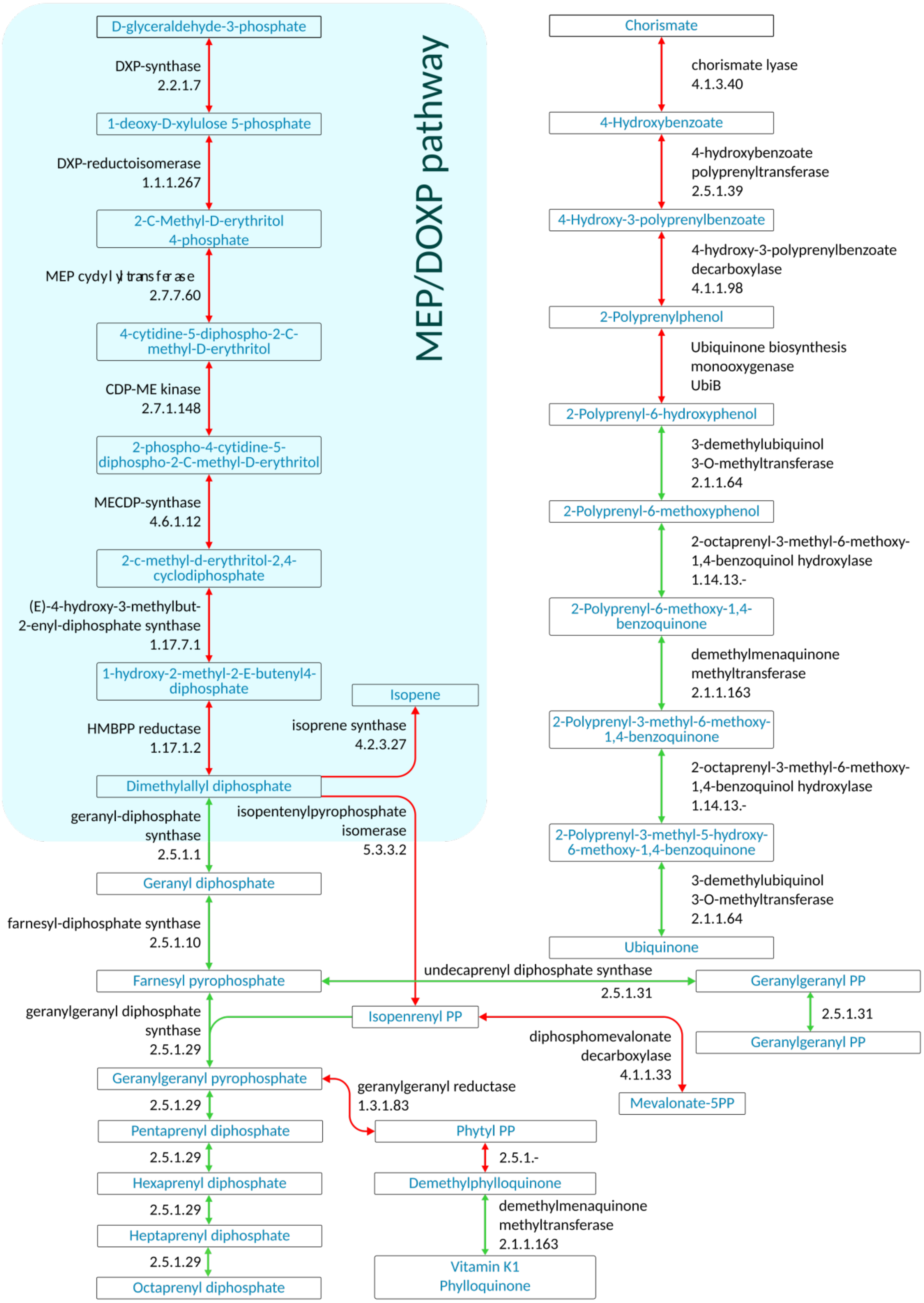

Supplementary Figure 6. Ubiquinone and vitamin K metabolism in *Holospora* spp. Red arrows represents missing enzymes
